# The effect of ginseng on sexual function in postmenopausal women with major depression: a triple-blind randomized controlled trial

**DOI:** 10.1186/s40780-025-00461-2

**Published:** 2025-06-18

**Authors:** Zahra Sharifpour, Shirin Hasanpoor, Sakineh Mohammad-Alizadeh-Charandabi, Zahra Mousavi, Elnaz Shaseb, Mojgan Mirghafourvand

**Affiliations:** 1 Master student of Midwifery, Department of Midwifery, School of Nursing and Midwifery, Tabriz University of Medical Sciences, Tabriz, Iran; 2 Physical Medicine and Rehabilitation Research Center, Tabriz University of Medical Sciences, Tabriz, Iran; 3 Department of Midwifery, School of Nursing and Midwifery, Tabriz University of Medical Sciences, Tabriz, Iran; 4 Department of Psychiatry, School of Medicine, Razi Hospital, Tabriz University of Medical Sciences, Tabriz, Iran; 5 Department of Clinical Pharmacy, School of Pharmacy, Tabriz University of Medical Sciences, Tabriz, Iran; 6 Social Determinants of Health Research Center, Department of Midwifery, Faculty of Nursing and Midwifery, Tabriz University of Medical Sciences, Tabriz, Iran

**Keywords:** Ginseng, Sexual function, Postmenopausal women, Major depression

## Abstract

**Background:**

Women often experience a decline in sexual desire as they age, particularly during menopause. An increase in sexual dysfunction is associated with the worsening of genitourinary symptoms that occur with menopause. Anxiety, fear, and depression in postmenopausal women may further deteriorate sexual dysfunction. Utilizing modern and effective methods to enhance sexual desire in these women is a priority in midwifery care. Given previous studies, ginseng is a herbal medicine that may be suitable in this regard. This study aimed to determine the effect of ginseng on sexual function (primary outcome), menopause symptoms, depression symptoms and side events (secondary outcomes) in postmenopausal women with major depression.

**Methods:**

This triple-blind randomized controlled trial was conducted on postmenopausal women with major depression in Tabriz, Iran between December 2022 and March 2024. A total of 66 postmenopausal women aged 45 to 60 with major depressive disorder were randomly assigned to intervention and control groups using block randomization. The intervention group received a 250-mg ginseng capsule twice daily after meals for eight weeks, while the control group received two gelatin placebo capsules (containing liquid edible paraffin) daily, similar in appearance to the ginseng capsules. Data collection was performed using the Female Sexual Function Index (FSFI), the Beck Depression Inventory (BDI), and the Greene Climactric Scale (GCS). The independent t-test and ANCOVA were used for data analysis.

**Results:**

The two groups did not show statistically significant differences in terms of demographic and baseline outcome measures. After the intervention, the mean overall sexual function score in the ginseng group was significantly higher than in the control group (adjusted mean difference (AMD): 2.17; 95% confidence interval (95%CI): 1.32 to 3.03, *P* = 0.001). The mean overall menopause symptoms score (AMD: -3.61; 95% CI: -5.47 to -1.74, *P* < 0.001) and depression score (AMD: -3.96; 95% CI: -5.76 to -2.20, *P* < 0.001) were significantly lower in the ginseng group compared to the placebo group.

**Conclusion:**

Ginseng is effective in improving sexual function and reducing menopause symptoms and depression in women with major depression. However, further research is needed to draw definitive conclusions.

**Trial registration:**

Iranian Registry of Clinical Trials (IRCT): IRCT20120718010324N74. Date of registration: 10/12/2022; URL: https://irct.behdasht.gov.ir/user/trial/65711/view; Date of first registration: 20/12/2022.

## Introduction

Natural menopause is defined as the permanent cessation of menstruation resulting from the loss of ovarian follicular activity, which is confirmed after 12 consecutive months of amenorrhea, with no other obvious pathological or physiological cause [1]. Menopause typically occurs at an average age of 52 years, within a range of 40 to 58 years [2]. In Iran, the average age of menopause is 48 years [3]. Emotional and psychological disorders are associated with earlier menopause [4]. Menopause is a natural event in a woman’s life, accompanied by many physical and emotional changes [5]. Menopausal symptoms include: 1) vasomotor symptoms (hot flashes, palpitations, night sweats, headaches) [6, 7], memory and learning disorders [8], mood and sleep disturbances [9, 10], urogenital syndrome, vaginal dryness, dyspareunia [6, 11], skin problems [12], decreased sexual function [13], and depression [14].

Women typically experience a decline in sexual desire as they age and during menopause [15]. Sexual disorders are a heterogeneous group of disorders that result from significant clinical disturbances in an individual’s ability to respond sexually or experience sexual pleasure [16]. The prevalence of sexual dysfunction among postmenopausal women aged 40 to 59 in the United States has been reported at 56.8% [17], and various studies indicate a range between 21% and 98.5% [13]. Among Iranian postmenopausal women aged 43 to 64, the prevalence has been reported as 69.8% in the domain of desire and 61.7% in the domain of arousal [18]. The presence of anxiety, stress, and depression in postmenopausal women exacerbates sexual dysfunction [19]. Major depressive disorder is correlated with sexual dysfunction [20]. Addressing sexual function in patients with major depression is one of the most important priorities in midwifery care [21].

The available treatments for managing menopausal symptoms include combined hormone therapy (increases the risk of breast cancer) [22], acupuncture (reduces some menopausal symptoms but does not affect hot flashes) [23], exercise and yoga (improves urogenital symptoms but have no significant effect on psychological, vasomotor, sexual symptoms, or quality of life scores) [24, 25], hypnosis [26], and stress management [27]. Herbal remedies such as licorice, valerian, soy, sage, and ginseng have been found to improve menopausal symptoms [26]. Ginseng, a plant from the Araliaceae family, has several reported benefits, including improvement of glucose metabolism [28], vasodilation and cardiovascular protection, treatment of cardiovascular disease [29], fatigue reduction, cancer prevention, and improvement of respiratory diseases [30]. However, some side effects of ginseng have been reported, including nausea, headache, palpitations, blood pressure fluctuations (either increase or decrease), insomnia [31], vaginal bleeding [32], and breast pain [33].

As women age and reach menopause, they frequently face a reduction in sexual desire and performance, which can be further intensified by symptoms like anxiety and depression. Ginseng is well-known for its potential to improve sexual function, reduce menopausal symptoms, and alleviate depression, particularly in women who have gone through menopause [34]. Several studies have shown that ginseng has a significant effect on improving sexual function [35, 36], enhancing quality of life [37], and reducing menopausal symptoms [38, 39]. The pharmacokinetics of ginseng have been extensively studied in humans, and when consumed in controlled amounts, it has almost no toxic effects [40]. In the existing literature, ginseng has been studied exclusively in postmenopausal women who experienced natural menopause, with no investigations focusing on surgical menopause or other menopause-inducing conditions. Given the prevalence of sexual dysfunction among patients with major depressive disorder and the absence of research on this specific population, this study addresses a critical gap. While some studies have examined the effects of ginseng in menopausal women, to our knowledge, this is the first triple-blind randomized controlled trial to evaluate its impact on sexual function in postmenopausal women with confirmed major depressive disorder. Therefore, this study was conducted to determine the effect of ginseng on sexual function (primary outcome), menopause symptoms, depression symptoms and side events (secondary outcomes) in postmenopausal women with major depression.

## Materials and methods

### Study type and participants

This triple-blind, randomized, controlled clinical trial (participants, researchers, and data analysts were unaware of the intervention) was conducted between December 2022 and March 2024. The study population included postmenopausal women with major depressive disorder who attended the Sheikh Al-Rais Specialized and Subspecialized Clinic affiliated with Tabriz University of Medical Sciences and the Psychiatry Clinic at Imam Reza Hospital.

The study participants were selected based on the following inclusion criteria: Age between 45 and 60 years old, having major depressive disorder confirmed by a psychiatrist, ability to read and write, having a stable sexual partner and engaging in sexual intercourse at least once a month, passing at least 12 months since the last menstrual period, a Female Sexual Function Index (FSFI) score of less than 28 [41], and no changes in the treatment plan in the past two months or expected in the next two months (including no current treatment, consistent use of antidepressants, or ongoing non-drug therapy). Exclusion criteria included: a history of ginseng consumption in the past three months, chronic diseases such as cardiovascular disease, liver disease, or diabetes, according to medical records, hormone therapy for any reason in the past two months, blood pressure above 140/90 mmHg, based on measurements by the researcher and medical records, continuous use of other herbal medicines, and use of medications that interact with ginseng, such as diabetes medications, immunosuppressants, and warfarin [28].

The sample size was calculated using G-Power software based on Ghorbani et al.‘s [38] findings on sexual function. The parameters considered were a mean of 21.73 for Group 1 (M_1_) and 15.99 for Group 2 (M_2_), with standard deviations of 5.45 (SD_1_) and 7.72 (SD_2_), respectively. A two-sided α of 0.05 and a power of 90% were used in the calculation, resulting in a required sample size of 30 participants per group. Accounting for a 10% dropout rate, the final sample size was adjusted to 33 participants per group.

### Sampling

Sampling for this study began after obtaining ethical approval from the Ethics Committee of Tabriz University of Medical Sciences (code IR.TBZMED.REC.1401.810) and registering the study with the Iranian Clinical Trials Center (IRCT20120718010324N74). The sampling method used was convenience sampling. The researcher visited the Sheikh-al-Rais Specialized and Subspecialized Clinic and the Psychiatry Clinic at Imam Reza Hospital, both affiliated with Tabriz University of Medical Sciences. Under the supervision of a psychiatric colleague, postmenopausal women with major depressive disorder were screened for eligibility based on inclusion and exclusion criteria. Those who met the criteria and consented to participate were asked to provide written informed consent. The socio-demographic characteristics questionnaire, the Female Sexual Function Index (FSFI), the Greene Climactric Scale (GCS), and the Beck Depression Inventory (BDI) were completed through interviews with the participants. Participants were followed up for eight weeks, and immediately after the intervention, the FSFI, the GCS, and the BDI were administered again.

### Randomization and intervention

Participants were randomly assigned to either the intervention group (receiving ginseng) or the control group (receiving a placebo) using block randomization with block sizes of four and six and a 1:1 allocation ratio. To ensure blinding, opaque, consecutively numbered bottles were used. At the start of the intervention, each participant was given a bottle containing 112 pills of either ginseng or placebo, according to their order of entry into the study, and were instructed on how to take the medication. Randomization and allocation concealment were performed by an individual who was not involved in sampling or data collection.

The medication and placebo were manufactured by Gol Darou Company in Isfahan-Iran. The intervention lasted for eight weeks, during which participants in the intervention group received 250 mg of ginseng twice daily after meals. Participants in the control group received two gelatin capsules of placebo daily, identical in appearance to the ginseng capsules, following the same dosing instructions. Participants were followed up regularly on a weekly basis via phone calls to emphasize the consistent medication use and to assess any side effects. It is important to note that all participants maintained their routine treatment for depression (primarily selective serotonin reuptake inhibitors (SSRIs)) throughout the study period, with no changes to medication type or dosage.

### Data collection tools

The data collection tools included a socio-demographic characteristics questionnaire, the Female Sexual Function Index (FSFI), the Greene Climactric Scale (GCS), the Beck Depression Inventory, an adverse events checklist, and a medication use checklist.

#### Socio-demographic characteristics questionnaire

This questionnaire, completed at the beginning of the study, included questions about age, age at menopause, duration of menopause, marital status, occupation, sufficiency of monthly income for living costs, education level, housing type, and number of living children.

*Female Sexual Function Index (FSFI)*: This questionnaire was designed by Rosen et al. in 2000 [42]. It consists of 19 questions across six domains: desire (2 items), arousal (4 items), vaginal lubrication (4 items), orgasm (3 items), satisfaction (3 items), and pain (3 items). Each item is scored from zero (or one) to five. The minimum score is 2, and the maximum score is 36. The psychometric properties of the Persian version of this questionnaire have been validated in Iran by Mohammadi et al., making the Persian FSFI a reliable and valid tool for assessing female sexual function, which can be used as a screening tool [41]. In the study by Ghorbani et al., the Cronbach’s alpha coefficient and intraclass correlation coefficient for the FSFI were reported as 0.94 and 0.74, respectively [38].

#### Greene climactric scale (GCS)

This scale is based on the standardized scale designed by Professor Greene to assess menopause symptoms. This scale includes 21 items that evaluate the presence and severity of menopause symptoms, including psychological (items 1–11), physical (items 12–18), vasomotor (items 19 and 20), and sexual symptoms (item 21). Each item is scored from zero (no symptoms) to 3 (very bothersome) based on the respondent’s experiences over the past four weeks. The total menopause symptoms score is calculated as the sum of scores across these four domains, with higher scores indicating more severe menopause symptoms. This tool has been used in numerous studies in Iran, and its validity and reliability have been confirmed. In Ghorbani et al.‘s study, the Cronbach’s alpha coefficient and intraclass correlation coefficient for this scale were reported as 0.79 and 0.99, respectively [38].

*Beck Depression Inventory (BDI)*: The 21-item BDI will be used to assess the depression levels of patients. This questionnaire was initially developed by Beck et al. in 1961. In 1996, Beck and his team made significant revisions to the inventory to cover a broader range of symptoms and to better align with the diagnostic criteria of depressive disorders as outlined in the Diagnostic and Statistical Manual of Mental Disorders (DSM-IV). The BDI is a self-report tool that takes approximately five to ten minutes to complete. It consists of 21 items related to various symptoms, which respondents rate on a four-point scale from zero to three. The items address aspects such as depression, pessimism, feelings of helplessness and failure, guilt, sleep disturbances, loss of appetite, self-loathing, and others. Respondents need to have at least a fifth or sixth-grade reading level to understand the items. Scores are calculated by summing the ratings for each item, with the minimum score being zero and the maximum being 63. The total score is used to determine the level of depression: 0 to 13 indicates no or minimal depression, 14 to 19 indicates mild depression, 20 to 28 indicates moderate depression, and 29 to 63 indicates severe depression. The validity and reliability of the BDI have been confirmed in the Iranian elderly population by Hamidi et al. with an intraclass correlation coefficient of 0.81 [43].

#### Adverse events checklist

In this checklist, any adverse events that occurred during the study and their severity were recorded.

#### Medication use checklist

Each participant recorded the daily consumption of ginseng or placebo after every intake in the medication use checklist.

### Data analysis

Data analysis was performed using SPSS version 26. The normality of quantitative data was assessed using the Kolmogorov-Smirnov test, and all data were found to be normally distributed. To compare socio-demographic characteristics between the two groups, Fisher’s exact test, chi-square for trend, and independent t-tests were used. To compare outcomes between the two groups before the intervention, an independent t-test was employed, and after the intervention, ANCOVA with baseline scores was used. All analyses were based on the intention-to-treat principle. A p-value of < 0.05 was considered statistically significant.

## Results

**Fig. 1:**
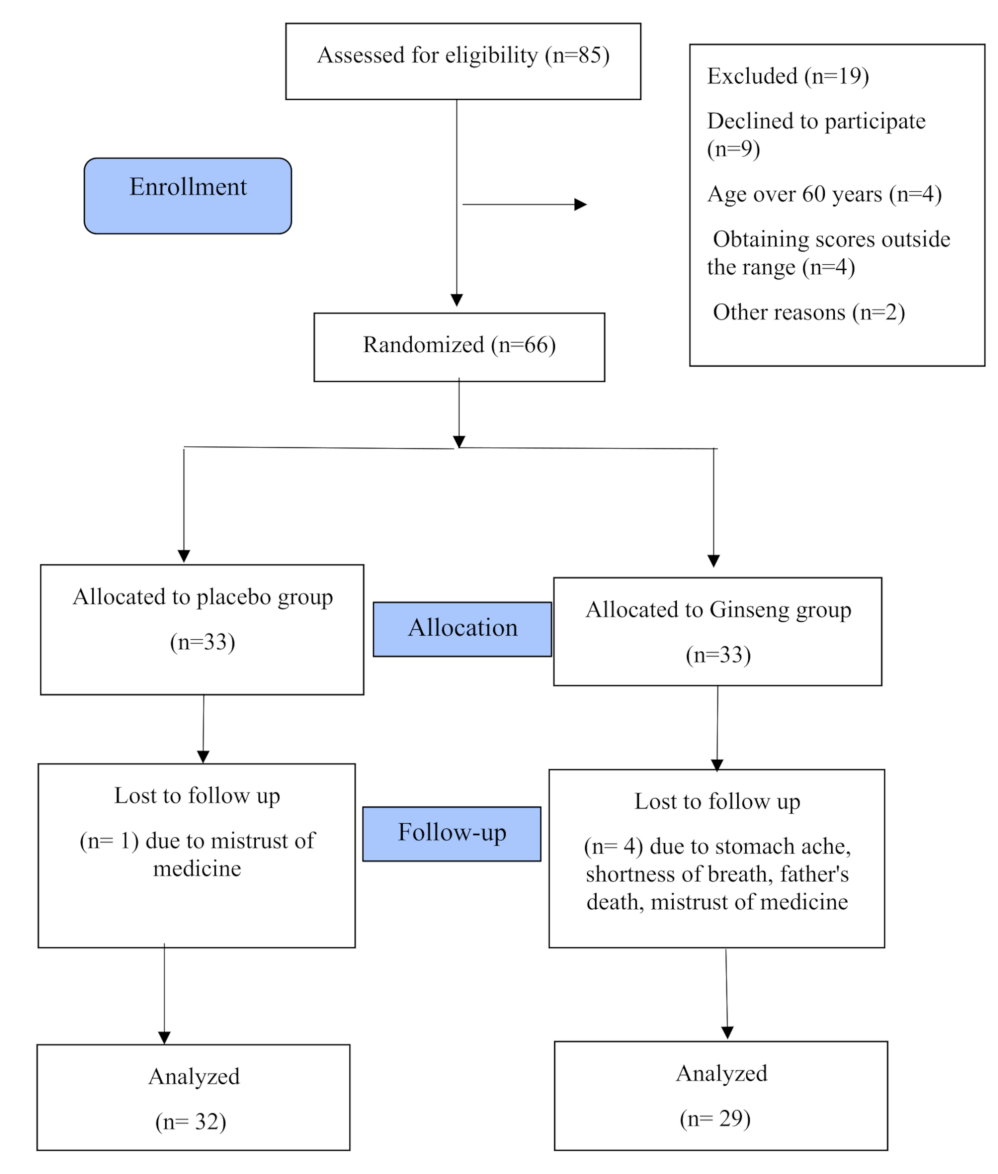
Study flow diagram

In this study, 72 patients were assessed to achieve the required sample size, of which six did not meet the inclusion criteria. Ultimately, 66 participants were divided between the two groups, and by the end of the study, five patients withdrew due to reasons such as discontinuation of cooperation, the death of relatives, and drug side effects (Fig. 1).

Table 1 shows the participants’ socio-demographic characteristics. The mean (SD) age and age at menopause were 51.78 (4.07) years and 48.12 (2.30) years, respectively, for the ginseng group and 51.18 (3.68) years and 47.33 (3.29) years for the placebo group. The majority of participants in the ginseng group (81.8%) and the placebo group (93.9%) were homemakers. More than one-third of women in the ginseng group (39.4%) and about half of the women in the placebo group (51.5%) had insufficient income. There were no statistically significant differences between the two groups in terms of socio-demographic characteristics (*p* > 0.05).

**Table 1:** The demographic characteristic of the participants by the study groups

Variable	Ginseng (*n* = 33) Mean (SD^d^)	Placebo (*n* = 33) Mean (SD^d^)	*P*-value
**Age** (Year)	51.8 (4.1)	51.2 (3.7)	0.529^a^
**Menopausal Age** (Year)	48.1 (2.3)	47.3 (3.3)	0.264^a^
**Menopausal duration** (Year)	3.4 (2.8)	3.9 (2.9)	0.492^a^
**Body Mass Index** (Kg/m^2^)	27.2 (3.7)	29.0 (4.9)	0.092^a^
**Sistolic blood pressure** (mmHg)	113.9 (12.0)	115.7 (9.9)	0.506^a^
**Diastolic blood pressure** (mmHg)	76.1 (8.5)	75.6 (8.8)	0.799^a^
	**Number (percent)**	**Number (percent)**	
**Occupation**			0.258^b^
Housewife	27 (81.8)	31 (93.9)	
Employed	6 (18.2)	2 (6.1)	
**Education**			0.411^c^
Underdiploma	22 (66.7)	24 (72.7)	
Diploma	8 (24.2)	8 (24.2)	
University	3 (9.1)	1 (3.0)	
**Spouse’s occupation**			0.177^b^
Unemployed	2 (6.1)	6 (18.2)	
Self-employed	14 (42.4)	13 (39.4)	
Worker	6 (18.2)	8 (24.2)	
Employed	6 (18.2)	3 (9.1)	
Retired	0 (0)	2 (6.1)	
Other	5 (15.2)	1 (3.0)	
**Sufficiency of income for living costs**			0.495^c^
Completely sufficient	8 (24.2)	4 (12.1)	
Relatively sufficient	13 (39.4)	17 (51.5)	
Insufficient	12 (36.4)	12 (36.4)	
**Housing type**			0.202^b^
Personal	29 (87.9)	25 (75.8)	
Rental	4 (12.1)	8 (24.2)	
**Number of children**			0.877^b^
Two and lower	20 (60.6)	19 (57.6)	
Three	10 (30.3)	9 (27.3)	
Four	3 (9.1)	5 (15.2)	

^a^ Independent t-test; ^b^ Fisher’s exact test; ^c^ Chi-square for trend; ^d^ Standard Deviation

After the intervention, significant differences were observed in the scores of all outcomes between the ginseng and placebo groups and the results highlight the effectiveness of ginseng in reducing depression and menopausal symptoms and improving sexual function in the study population.

The mean (SD) total score of the FSFI in the ginseng group increased from 10.64 (SD: 0.90) before the intervention to 13.19 (SD: 1.93) after the intervention. The placebo group, however, showed a change from from 10.30 (SD: 0.76) to 10.80 (SD: 1.71). The adjusted mean difference (AMD) for the FSFI scores was 2.17 (95% CI: 1.32 to 3.03; *p* < 0.001), indicating a significant enhancement in the ginseng group compared to the placebo group. Regarding the subscales of sexual function, there was no significant statistical difference between the study groups before the intervention. However, after the intervention, the mean scores for all subscales except of lubrication were significantly higher in the ginseng group compared to the placebo group (*p* < 0.05) (Table 2).

**Table 2:** Comparison of the mean score of sexual function and its subdomains among the study groups

Variable	Ginseng		Placebo		MD (95% CI)^b^	*P*-value
	*N*	Mean (SD^a^)	*N*	Mean (SD^a^)		
**Overall score of sexual function (scores 2 to 36)**						
Before intervention	33	10.64 (0.90)	33	10.30 (0.76)	0.34 (-3.14 to 1.10)	0.109^c^
After intervention	32	13.19 (1.93)	29	10.80 (1.71)	2.17 (-1.32 to 3.03)	< 0.001^d^
**Desire (score range: 2 to 10)**						
Before intervention	33	1.94 (0.60)	33	1.89 (0.37)	0.04 (-0.19 to 0.30)	0.659^c^
After intervention	32	2.51 (0.65)	29	2.13 (0.61)	0.38 (0.06 to 0.71)	0.022^d^
**Arousal (score range: 0 to 20)**						
Before intervention	33	1.46 (0.22)	33	1.47 (0.21)	-0.01 (-0.11 to 0.09)	0.867^c^
After intervention	32	2.12 (0.44)	29	1.45 (0.32)	0.66 (0.46 to 0.87)	< 0.001^d^
**Lubrication (score range: 0 to 20)**						
Before intervention	33	1.82 (0.39)	33	1.78 (0.30)	0.04 (-0.12 to -0.21)	0.603^c^
After intervention	32	1.96 (0.50)	29	1.76 (0.41)	0.19 (-0.04 to 0.43)	0.110^d^
**Orgasm (score range: 0 to 15)**						
Before intervention	33	1.43 (0.26)	33	1.39 (0.26)	0.04 (-0.09 to 0.16)	0.581^c^
After intervention	32	1.86 (0.42)	29	1.51 (0.34)	0.34 (0.14 to 0.54)	0.001^d^
**Satisfaction (score range: 0 to 15)**						
Before intervention	33	1.85 (0.54)	33	1.58 (0.43)	0.26 (0.01 to 0.51)	0.042^c^
After intervention	32	1.86 (0.52)	29	1.56 (0.40)	0.38 (0.06 to 0.71)	0.041^d^
**Pain (score range: 0 to 15)**						
Before intervention	33	2.18 (0.84)	33	2.87 (0.72)	-0.19 (-0.61 to 0.22)	0.360^c^
After intervention	32	2.87 (0.72)	29	2.34 (0.80)	0.48 (0.10 to 0.87)	0.014^d^

Higher scores indicate more sexual performance

^a^ Standard Deviation; ^b^ Mean Difference (95% Confidence Interval); ^c^ independent t-test; ^d^ ANCOVA test adjusted for baseline score

The mean total score for menopausal symptoms in the ginseng group decreased from 33.90 (SD: 6.06) before the intervention to 29.71 (SD: 6.16) after the intervention. The placebo group, however, showed a decrease from 35.57 (SD: 6.42) to 34.41 (SD: 6.53). The AMD for menopausal symptoms scores was − 3.61 (95% CI: -5.47 to -1.74; *p* < 0.001), demonstrating a significant improvement in the ginseng group compared to the placebo group. There was no significant statistical difference between the study groups regarding the Greene subscales before the intervention. However, after the intervention, the mean scores for all subscales in the ginseng group were significantly lower than those in the placebo group (*p* < 0.05) (Table 3).

**Table 3:** Comparison of the average overall score of menopause symptoms and its subdomains among the study groups

Variable	Ginseng		Placebo		MD (95% CI)^b^	*P*-value	
	*N*	Mean (SD^a^)	*N*	Mean (SD^a^)			
**Overall score of menopause symptoms (score range: 0 to 63)**							
Before intervention	33	33.90 (6.06)	33	35.57 (6.42)	-1.67 (-4.74 to 1.40)	0.283^c^	
After intervention	32	29.71 (6.16)	29	34.41 (6.53)	-3.61 (-5.47 to -1.74)	< 0.001^d^	
**Psychological symptoms (score range: 0 to 33)**							
Before intervention	33	21.54 (2.81)	33	22.06 (3.18)	-0.51 (-1.99 to 0.96)	0.489^c^	
After intervention	32	19.37 (3.54)	29	21.34 (3.46)	-1.80 (-3.07 to -0.52)	0.006^d^	
**Physical symptoms (score range: 0 to 21)**							
Before intervention	33	6.63 (3.57)	33	7.66 (3.53)	-1.03 (-2.77 to 0.71)	0.243^c^	
After intervention	32	5.96 (3.44)	29	7.27 (3.49)	-0.41 (-0.88 to 0.05)	0.080^d^	
**Vasomotor symptoms (score range: 0 to 6)**							
Before intervention	33	4.18 (1.21)	33	4.19 (1.15)	0.0 (-0.58 to 0.58)	1.00^c^	
After intervention	32	3.21 (1.0)	29	4.17 (1.13)	-0.88 (-1.30 to -0.46)	0.001^d^	
**Sexual symptoms (score range: 0 to 3)**							
Before intervention	33	1.54 (1.32)	33	1.66 (1.40)	-0.12 (-0.79 to 0.55)	0.720^c^	
After intervention	32	1.15 (1.22)	29	1.62 (1.44)	-0.44 (-0.80 to -0.08)	0.017^d^	

Higher scores indicate more severe menopausal symptoms.

^a^ Standard Deviation; ^b^ Mean Difference (95% Confidence Interval); ^c^ independent t-test; ^d^ ANCOVA test adjusted for baseline score

The mean depression score in the ginseng group decreased from 50.90 (SD: 5.06) before the intervention to 46.03 (SD: 6.10) after the intervention. In contrast, the placebo group showed a decrease from 51.42 (SD: 4.95) to 49.86 (SD: 5.46). The AMD for depression scores was − 3.98 (95% CI: -5.76 to -2.20; *p* < 0.001), indicating a significant reduction in the ginseng group compared to the placebo group (Table 4).

**Table 4:** Comparison of the average score of depression among the study groups

Variable	Ginseng		Placebo		MD (95% CI)^b^	*P*-value
	*N*	Mean (SD^a^)	*N*	Mean (SD^a^)		
**Depression (score range: 0 to 63)**						
Before intervention	33	50.90 (5.06)	33	51.42 (4.95)	-0.51 (-2.97 to 1.94)	0.678^c^
After intervention	32	46.03 (6.10)	29	49.86 (5.46)	-3.98 (-5.76 to -2.20)	< 0.001^d^

^a^ Standard Deviation; ^b^ Mean Difference (95% Confidence Interval); ^c^ independent t-test; ^d^ ANCOVA test adjusted for baseline score

No side event has been reported by the ginseng group. However, in the placebo group, one participant reported stomach ache and another participant reported shortness of breath.

## Discussion

The present study indicated that ginseng significantly affected sexual function, menopausal symptoms, and depression.

After the intervention, the average sexual function score in the ginseng group was significantly higher compared to the placebo group. A study by Asgharpour et al. (2021) aimed at determining the effects of oral ginseng on erectile dysfunction and fatigue in prostate cancer patients found that oral ginseng improved erectile dysfunction, reduced fatigue, and ultimately enhanced quality of life indicators. However, attention should be paid to the treatment duration, potential interactions, and possible side effects of this supplement [44]. Ghamari et al. conducted a clinical trial to determine the effect of vitamin E and ginseng supplementation on sexual enhancement in women. Their results showed that the average sexual function score in menopausal women receiving both ginseng and vitamin E was significantly higher than in the control group [45]. A randomized clinical trial by Ghorbani et al. (2018) in Tabriz-Iran demonstrated that after the intervention, the average sexual function score in the ginseng group significantly increased compared to the placebo group [38]. Another study by OH et al., aimed at determining the effect of ginseng on sexual arousal in menopausal women, found that the average scores for arousal and satisfaction were significantly higher in the ginseng group compared to the placebo group [39]. All the studies above align with the present study, indicating the positive effect of ginseng on sexual function. It appears that the primary mechanism by which ginseng improves sexual function is through modulation of the nervous and hormonal systems, acting directly on hormonal receptors, which enhances sexual function in both genders [46].

In this study, after the intervention, the average score of menopausal symptoms in the ginseng group was significantly lower than in the placebo group. A randomized controlled trial conducted by Chung et al. (2021) in South Korea aimed at determining the effect of red ginseng on menopausal symptoms in post-surgical breast cancer patients revealed that after the intervention, all dimensions of menopausal symptoms improved in the ginseng group [47]. Another randomized controlled trial by Ghorbani et al. (2018) in menopausal women aged 45–60 in Tabriz-Iran showed that after the intervention, all dimensions of menopausal symptoms improved in the ginseng group [38]. A randomized controlled trial by Kim et al., aimed at assessing the “effect of red ginseng on menopausal symptoms and cardiovascular risk factors” in menopausal women, found that after the intervention, there was a significant difference in menopausal symptoms between the two groups. The mean score for hot flashes, as measured by the Cooper Menopause Index and MRS score, significantly decreased in the intervention group compared to the placebo group [48]. The mechanisms through which ginseng might affect menopausal symptoms are thought to include estrogen-like hormonal effects. Ginsenosides, the main active components of ginseng, have been shown to exert estrogen-like effects without directly binding to estrogen receptors [49].

In this study, after the intervention, the average depression score in the ginseng group was significantly lower than in the placebo group. A randomized controlled trial by Lee et al. (2017) in South Korea aimed at assessing the effect of ginseng on depression and anxiety caused by stress in patients with hypothalamic disorders found that after the intervention, the average depression score in the ginseng group improved across all dimensions [50]. A systematic review by Kim and Cho (2021) in South Korea on the effects of ginsenosides on depression in preclinical studies indicated that ginsenosides, including ginsenoside Rg1, reduce depressive symptoms, modulate underlying mechanisms, and could be a promising antidepressant [51]. Tavakoli and Hassan Zadeh Khush (2024) conducted a semi-experimental study to determine the effect of 8 weeks of resistance training combined with ginseng consumption on anxiety and depression in older women. In this study, 30 eligible elderly individuals were randomly assigned to three groups: low-volume resistance training with ginseng, high-volume resistance training with ginseng, and a control group (10 individuals). The results showed that in both ginseng intervention groups, there was a significant difference in depression between pre-test and post-test, while no significant difference was observed in the control group [52]. Ginseng effectively regulates the immune response and hormonal changes due to stress, thereby maintaining homeostasis. Besides suppressing the onset of mental disorders such as anxiety and depression, ginseng also prevents stress-related physiological diseases. Ginseng plays a role in regulating the hypothalamic-pituitary-adrenal axis and controlling hormones, thus having beneficial effects on the heart, brain, and bone diseases and reducing erectile dysfunction. Recent studies have highlighted the potential use of ginseng in the prevention and treatment of chronic inflammatory diseases such as diabetes, rheumatoid arthritis, and allergic asthma. However, the underlying mechanisms of ginseng’s effects on these stress-related diseases are not yet fully established [50].

The results of this research support the idea that ginseng is beneficial for enhancing sexual function, alleviating menopausal symptoms, and mitigating depression in women diagnosed with major depressive disorder. The ways in which ginseng brings about these effects may involve the modulation of the nervous and hormonal systems, directly affecting hormonal receptors and thereby improving sexual function in both sexes. Additionally, ginsenosides, the active compounds found in ginseng, are thought to mimic estrogen effects, potentially easing menopausal symptoms without directly interacting with estrogen receptors. This dual benefit of enhancing sexual function while also addressing menopausal symptoms and depression presents ginseng as a promising herbal treatmentfor postmenopausal women facing these difficulties [53].

The results of this study suggest that ginseng may be considered as a complementary treatment for postmenopausal women with major depression experiencing sexual dysfunction and menopausal symptoms. It may provide a safer alternative or adjunct to conventional therapies. Nevertheless, it is crucial to acknowledge that although the findings are encouraging, further studies with larger sample sizes and more rigorous methodologies are needed to establish firm conclusions about the efficacy of ginseng in these domains. The results of this study can inform future educational initiatives and interventions aimed at improving the health and quality of life for women suffering from major depressive disorder.

### Strengths and limitations

One of this study’s strengths is its adherence to all principles of clinical trials, including random allocation, allocation concealment and blinding. Another strength is the use of standardized tools for outcome measurement. However, a limitation of this study is that the post-test was conducted only once after the medication was completed. It is recommended that future studies explore long-term effects as well.

## Conclusion

Ginseng is effective in improving sexual function and reducing menopause symptoms and depression in women with major depression. Additional research with stronger evidence is necessary to draw firm conclusions regarding the effects of ginseng on sexual function, menopausal symptoms, and depression in women suffering from major depressive disorder. This population is particularly susceptible and in need of assistance. Timely and appropriate measures should be taken to support this group of women. Therefore, the findings of this study can guide educational efforts toward improving the health of women with major depressive disorder.

## Data Availability

The datasets generated and/or analyzed during the current study are not publicly available due to limitations of ethical approval involving the patient data and anonymity but are available from the corresponding author at reasonable request.
